# On Albumen in the Urine after Scarlatina

**Published:** 1854-04

**Authors:** H. Bence Jones


*Medical Times and Gazette.*

*On Albumen in the Urine after Scarlatina.*

By Dr. H. Bence Jones, F. R. S., &c.

The question of the passage of the scarlet-fever poison out of the system through the kidneys, has still to be proved. The general treatment differs in no respect from that of Bright's disease. Except that recovery is more frequent, and that the symptoms yield more easily to treatment, I know of no difference between this disease and Bright's disease. Mercurials are as dangerous in the dropsy after scarlet fever, as in Bright's disease. Diuretics are as much to be avoided; sudorifics and aperients are as beneficial. In extreme cases of both diseases, the benefit of elaterium might be illustrated by many examples if my time admitted me to read them. In both diseases the blood becomes impure, from the accumulation of the products of the organic changes in the body. I might sum up the objects to be attained, if possible, in these few words, viz., relieve congestion, and purify the blood. The appearance of albumen in urine in cholera is caused by passive congestion of the kidney; while the albumen in rheumatic fever, is the result of active congestion. The albumen after the scarlet fever may also, at least as regards its treatment, be considered as produced by a state of more or less active congestion of the kidney.—*Braithwaite's Retrospect, from Medical Times and Gazette.*

